# Stemness Related Genes Revealed by Network Analysis Associated With Tumor Immune Microenvironment and the Clinical Outcome in Lung Adenocarcinoma

**DOI:** 10.3389/fgene.2020.549213

**Published:** 2020-09-16

**Authors:** Hao Zeng, Jianrui Ji, Xindi Song, Yeqian Huang, Hui Li, Juan Huang, Xuelei Ma

**Affiliations:** ^1^State Key Laboratory of Biotherapy and Cancer Center, West China Hospital, Sichuan University, Collaborative Innovation Center for Biotherapy, Chengdu, China; ^2^West China School of Medicine, West China Hospital, Sichuan University, Chengdu, China; ^3^Department of Hematology, Sichuan Academy of Medical Sciences and Sichuan Provincial People’s Hospital, University of Electronic Science and Technology of China, Chengdu, China

**Keywords:** cancer stemness cells (CSCs), tumor microenvironment (TME), clinical outcome, single cell RNA sequencing (scRNA-seq), weighted gene co-expression network analysis (WGCNA)

## Abstract

Lung adenocarcinoma (LUAD) is one of the leading fatal malignancy with high morbidity and mortality worldwide. However, due to its complicated mechanism and lack of effective clinical therapeutics, early diagnosis and prognosis are still unsatisfactory. Most of the previous studies focused on cancer stem cells (CSCs), the relationship between cancer stemness (stem-like characteristics) and anti-tumor immunity has not been clearly revealed. Therefore, this study aimed to comprehensively analyze the role of cancer stemness and tumor microenvironment (TME) in LUAD using weighted gene co-expression network analysis (WGCNA). We constructed a gene co-expression network, identified key modules, and hub genes, and further explored the relationship between hub gene expression and cancer immunological characteristics through a variety of algorithms, including Estimation of STromal and Immune cells in MAlignant Tumor tissues using Expression data (ESTIMATE) and Gene Set Enrichment Analysis (GSEA). The hub genes were renamed stemness related genes (SRGs), whose functions were examined at the transcription and protein levels through survival analysis with additional samples, Oncomine database, immunohistochemistry, single cell RNA sequencing (scRNA-seq) and single-sample Gene Set Enrichment Analysis (ssGSEA). Subsequently, Tumor Immune Dysfunction and Exclusion (TIDE) and Connectivity Map (CMap) were implemented for treatment and prognosis analyses. As a result, 15 co-expressed SRGs (CCNA2, CCNB1, CDC20, CDCA5, CDCA8, FEN1, KIF2C, KPNA2, MCM6, NUSAP1, RACGAP1, RRM2, SPAG5, TOP2A, and TPX2) were identified. The overexpression of which was discovered to be associated with reduced immune infiltration in LUAD. It was discovered that there was a general negative correlation between cancer stemness and immunity. The expression of SRGs could probably affect our tumor occurrence, progression, the efficacy of chemotherapy and immunotherapy, and clinical outcomes. In conclusion, the 15 SRGs reported in our study may be used as potential candidate biomarkers for prognostic indicators and therapeutic targets after further validation.

## Introduction

Lung cancer is one of the most widespread and lethal diseases that plague the global population. It accounts for 11.6% of new cases and 18.4% of deaths annually, which remains the highest among males (Bray et al., 2018). According to the pathological classification of lung cancer, non-small-cell lung cancer (NSCLC) constitutes approximately 80% of all cases (Barlesi et al., 2016). As the most prevalent type of NSCLC, lung adenocarcinoma (LUAD) is more common among non-smokers (especially women), accounting for 38.5% of all lung cancer cases (Dela Cruz et al., 2011). Its abnormal appearance in non-smokers inspired researchers to investigate the underlying risk factors. In the past few decades, the incidence of LUAD in both genders has increased much faster than that of squamous cell carcinoma. Since the 1970s, LUAD in men in the United States has almost doubled, and that in women has risen from approximately 25 to 33% (Ridge et al., 2013). Despite years of efforts to improve clinical outcomes with therapeutic strategies including surgery, radiotherapy, chemotherapy, immunotherapy, the prognosis is still less than satisfactory. Recently, targeted therapy based on its molecular alterations has been successfully applied to a variety of cancers, such as breast cancer (Xuhong et al., 2019), ovarian cancer (Aust et al., 2020), NSCLC (Santarpia et al., 2013), which encourages us to seek for more biomarkers that may serve as targeted agents.

In recent years, the researches of cancer stem cells (CSCs) have shown promising value (Friedmann-Morvinski and Verma, 2014; Shibue and Weinberg, 2017). CSCs are defined as the cells with stem cell-like characteristics in cancer, which have the ability to drive tumor formation and growth, and influence tumor progression and prognosis (Reya et al., 2001). Stemness represents the characteristics of self-renewing, differentiation from the cell of origin, and the ability to generate other types of cells in particular tissues. In a previous study, stemness index was introduced to assess the degree of oncogenic dedifferentiation by a machine-learning algorithm picking epigenetic and transcriptomic feature sets derived from non-transformed stem cells and the differentiated progeny of them, including RNA-based stemness index (mRNAsi), DNA methylation-based stemness index (mDNAsi), and epigenetically regulated-mRNAsi (EREG-mRNAsi) (Malta et al., 2018). In addition to cancer progression, increased stemness index was found in metastatic tumors and was associated with intratumoral heterogeneity, which may explain its association with tumor progression, staging, treatment resistance and poor prognosis (Shibue and Weinberg, 2017). Nevertheless, the relationship between cancer stemness (stem-like characteristics) and anti-tumor immunity has not been clearly revealed.

There is increasing evidence that tumor progression and prognosis are not only affected by genetic changes (such as stem cell-like characteristics), but also by the tumor microenvironment (TME) (Liu et al., 2018; Miranda et al., 2019). According to a previous study, it was concluded that the stemness index was related to the content of TME, and genetic variation in cancer cells may indirectly affect anti-tumor immunity (Malta et al., 2018). Some studies have confirmed that the occurrence and progression of tumors can be regulated by immune cells and factors in TME, which become promising targets for treatment and the basis of immunotherapy (Elinav et al., 2013; Topalian et al., 2016; Nagarsheth et al., 2017). For instance, blockers of programmed cell death protein 1 (PD1) and cytotoxic T-lymphocyte-associated antigen 4 (CTLA4) indicate that there are broad and diverse opportunities for enhancing anti-tumor immunity by modulating the immune responses (Pardoll, 2012). Therefore, it is necessary to supplement the research on the potential mechanism of CSCs and further apply them in treatments, especially the treatments related to immunity.

As a bioinformatics method in the field of medical data processing, weighted gene co-expression network analysis (WGCNA) has been used in numerous kinds of cancers, such as prostate cancer (Wang et al., 2009) and breast cancer (Presson et al., 2011). On this basis, we can describe the correlation between gene expression in different samples and identify candidate co-expressed genes or targets (Langfelder and Horvath, 2008). In this study, WGCNA was conducted to identify the potential gene modules, from which the key modules and genes related to cancer stemness were selected. The key genes in the chosen module were further validated by a variety of approaches. The expression of which was found to be related to cancer prognosis, staging, epithelial-mesenchymal transition (EMT) and reduced immune infiltration. The results also suggested that the efficacy of chemotherapy and immunotherapy could be different from the differential expression of key genes.

## Materials and Methods

### Database, Stemness Index, and Immune Score

In this study, we obtained the RNA-sequencing (RNA-seq) data of 533 LUAD samples and clinical characteristics of 522 LUAD samples from The Cancer Genome Atlas (TCGA) database^1^. The TCGA database demonstrates the landscape of primary tumors by forming the integrated molecular profiles comprising genomic, transcriptomic, epigenomic, and post-translational proteomic characteristics, as well as histopathological and clinical annotations. A total of 507 LUAD patients with available transcriptome profiling and clinical data were finally retained.

Gene expression datasets (GSE68465, GSE68571, and GSE69405) were downloaded from the Gene Expression Omnibus (GEO) database^2^. Among them, datasets of GSE68465 and GSE68571 were used for verifying the role of hub genes in clinical outcomes, and the single cell RNA sequencing (scRNA-seq) dataset GSE69405 was used to explore the relationship between hub genes and tumor-related signaling pathways.

The stemness index (mRNAsi index) was obtained from the attachment of the previous article (Malta et al., 2018), which was calculated by a one-class logistic regression (OCLR) machine learning algorithm (Sokolov et al., 2016) and was quantified as mRNAsi using a combination of multi-platform analysis. In addition, the immune cell infiltration was quantified using Estimation of STromal and Immune cells in MAlignant Tumors using Expression data (ESTIMATE) algorithm (Yoshihara et al., 2013), which possesses specific and sensitive discrimination of immune cells and is able to compute the immune score representing the proportion of tumor-infiltrating lymphocytes (TILs) in tumor tissues.

### Survival Analysis

For prognostic comparisons, the overall survival was analyzed enrolling 498 LUAD samples with stemness index, immune score and hub gene expression. The “surv_cutpoint” function in R package “survminer” was used to find the best cut-off points of continuous variables (i.e., mRNAsi, ImmuneScore and score of SRGs). In this study, we divided the patients into the high-value group or the low-value group using the best cut-off value of each variable.

### Construction of the Co-expression Network

The co-expression network was constructed using the R package WGCNA (Langfelder and Horvath, 2008). Genes having the highest 50% of variance were extracted to guarantee the accuracy and heterogeneity of bioinformatics statistics for following co-expression network analysis. The co-expression similarity matrix was formed by absolute values of correlations among transcription levels. We modified the Pearson correlation matrix for the paired genes. The definition of co-expression similarity (*s*_*i,j*_) was the absolute value of the correlation coefficient between the profiles of nodes *i* and *j*. It was calculated as follows:

$$s_{i,j}=|\text{cor}\left(x_{i},x_{j}\right)|a_{i,j}=s_{i,j}^{\beta }\left(\beta \geq 1\right)$$

Here, *x*_*i*_ and *x*_*j*_ represented a series of the expression value for gene *i* and *j*. Pearson’s correlation coefficient of gene *i* and *j* was represented by cor. Weighed network adjacency was further defined by raising co-expression similarity to power β. The adjacency coefficient (*a_*i*_,_*j*_*) was calculated to reflect the correlation between each pair of nodes. An appropriate β value of five (scale-free *R*^2^ = 0.95) was chosen as the soft-threshold parameter to improve the similarity of the matrix and to bring about a scale-free co-expression network. Modules were further spotted by hierarchical clustering of the weighting coefficient matrix. Additionally, the module membership of gene *i* in module *q* was defined as:

$$K\,\text{cor},i\,\left(q\right):=\text{cor}\,\left(x_{i},E\,\left(q\right)\right)$$

The *x*_*i*_ represented the profile of gene *i*. *E*(*q*) represented the module eigengene which was the principal member of an individual module of module *q*. Additionally, the module membership measured *K*(*q*) distributes in [−1, 1] and stood for the closeness of gene *i* to module *q*, *q* = 1,…, *Q*.

### Identification of Key Module and Hub Genes

In order to determine the stemness related module, the genetic significance (GS), module significance (MS) and module eigengenes (MEs) were introduced. GS was the *p*-value in the linear regression between gene expression and clinical data, and was converted into the log10 version of it (GS = lgp). The average value of GS was defined as MS, which represented the consistency of module and sample characteristics. Module eigengene was the superiority of the component in each module. The overall gene expression was represented by the feature expression profile of a specific module. The statistical significance depended on the corresponding *p*-value.

Afterward, GS and module membership (MM) were calculated for each gene in the blue module. MM was defined as the correspondence between genes and expression profiles. We chose the cut-off criteria of cor.gene MM > 0.8 and cor.gene GS > 0.5 to screen the hub genes. Then the hub genes were renamed stemness related genes (SRGs).

### Validation of Stemness Related Genes (SRGs)

We systematically examined the expression distribution and prognostic performance of SRGs at the transcription and protein levels. The performance of SRGs could be studied through R package Gene Set Variation Analysis (GSVA), which took a gene-by-sample expression matrix as an input and outputs a gene set-by-sample enrichment score matrix.

Initially, a total of 432 LUAD samples in GSE68465 and 86 LUAD samples in GSE68571 from GEO database were additionally used to verify the prognostic performance of SIRG signature. The Oncomine database^3^ allowed us to analyze the degree of difference between the expression of SRGs in LUAD tissues and normal tissues. Survival analysis was performed again to evaluate the correlation between SRGs expression and clinical characteristics and to compare it with the overall survival rate predicted by the stemness index and immune score, respectively. Besides, we evaluated the relationship between the SRGs and tumor staging and serval important tumor related signaling pathways and signatures. The validation of SRGs at the protein level was achieved by immunohistochemistry of SRGs based on the images in the Human Protein Atlas database.

To verify the relationship between SRGs and anti-tumor immunity, we compared the hematoxylin and eosin (H&E) histopathological images of TILs in LUAD samples between high and low SRGs expression groups. The H&E histopathological images were downloaded from the Cancer Imaging Archive (TCIA) portal^4^ and were further processed by Openslide Python library (Goode et al., 2013). Besides, the enrichment analysis of the key module was performed through Metascape^5^. In order to study the potential molecular biological mechanisms of the SRGs, we then downloaded data from the Molecular Signatures Database and performed gene-set enrichment analysis (GSEA) analysis.

### Inference of Infiltrating Cells in TME

The ESTIMATE algorithm (Yoshihara et al., 2013) was applied to explore the immune function of SRGs in term of immune responses. The score calculated by this algorithm represented the proportion of immune infiltration in tumor tissues. Subsequently, the infiltration levels of immune cells were assessed by Single-Sample Gene Set Enrichment Analysis (ssGSEA) (Bidkhori et al., 2013). It generated a single value to qualify the extent to which a gene set is coordinately up or down-regulated in a single sample (Barbie et al., 2009). A total of 24 immune phenotypes were involved in our study, including B cells, T cells, natural killer (NK) cells, dendritic cells (DCs), macrophages, and other TILs.

### Potential Application of SRGs With Clinical Therapeutic Strategies

The clinical value of SIRG was assessed through subclass mapping and Tumor Immune Dysfunction and Exclusion (TIDE) algorithm (Hoshida et al., 2007; Jiang et al., 2018). According to the pharmacogenomics database [the Genomics of Drug Sensitivity in Cancer (GDSC)^6^ ], five first-line medications for LUAD treatment (Cisplatin, Docetaxel, Gemcitabine, Paclitaxel, Vinorelbine) were selected, and the effect of chemotherapy was predicted by R package “pRRophetic.” The ridge regression was used to calculate the half-maximal inhibitory concentration (IC_50_) of each sample, then to assess the precision of prediction.

Moreover, we used the connectivity map (CMap) analysis and mechanisms of action (MoA) database to predict the relationship between small molecular inhibitors usage and SRGs in LUAD. CMap is an online pharmacogenomic database cataloging gene expression data from cultured cells treated individually with various chemicals, including a variety of phytochemicals. It connected small molecule drugs with diseases via gene-expression signatures, and each medicine was profiled by different cell lines. The connectivity score was adopted to estimate the degree of connectivity between the compound and the query signature with a range of −1 to 1. A positive score indicates that an agent might facilitate the expression of query signature, while a negative score implies that a drug might repress or reverse the expression trends of gene signature.

In this study, we uploaded the differentially expressed genes (DESeq2, adjusted *p* < 0.05 and | log2 fold-change| >1.5) between high and low SIRG group to the connectivity map website^7^. Combined with the mechanisms of action (MoA) database, specific small molecular compounds with negative connectivity enrichment scores were selected as potential therapeutic molecules to the LUAD patients with highly expressed SRGs.

## Results

### Significant Correlation of Stemness Index, Immune Score, and Clinical Outcome

**FIGURE 1:**
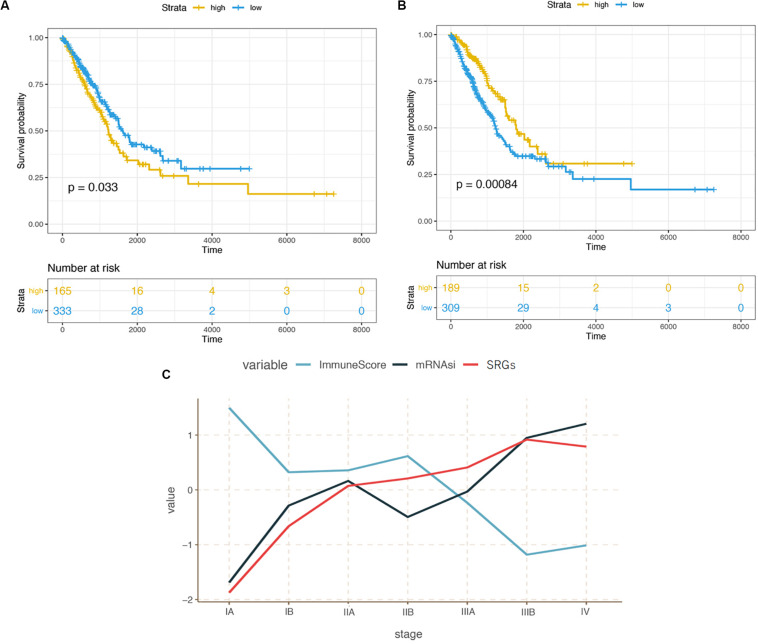
**(A)** Kaplan-Meier curves illustrating the relationship between cancer stemness and survival probability. **(B)** Kaplan-Meier curves illustrating the relationship between immunity infiltration and survival probability. **(C)** Correlation between stemness, immunity, expression of stemness related genes (SRGs), and the progression of LUAD from stage I to IV.

We downloaded the transcriptome dataset and clinical information of LUAD samples from the TCGA database, including gender, age, life-status, survival time and Tumor-Node-Metastasis (TNM) stage classification. The mRNAsi score (stemness index) was obtained from a previous study (Malta et al., 2018) based on the OCLR machine-learning algorithm (Sokolov et al., 2016). Here, mRNAsi was used to describe the similarity between tumor tissue and stem cells based on the gene expression. As shown in Figure 1A, compared with lower mRNAsi score group, patients with higher mRNAsi scores have shorter overall survival (*p* = 0.033), suggesting that high stemness index could be a risk factor for LUAD patients.

The correlation between tumor-related immunity and clinical performance was demonstrated in Figure 1B. Contrary to the survival analysis of mRNAsi scores, people with high infiltration levels showed significantly better outcomes than those with low infiltration levels in TME (*p* < 0.001). In term of staging, mRNAsi scores increased with the progression of cancer from early to advanced stage while the immune scores changed oppositely (Figure 1C).

### WGCNA: Identification of the Stemness Related Module and Genes

**FIGURE 2:**
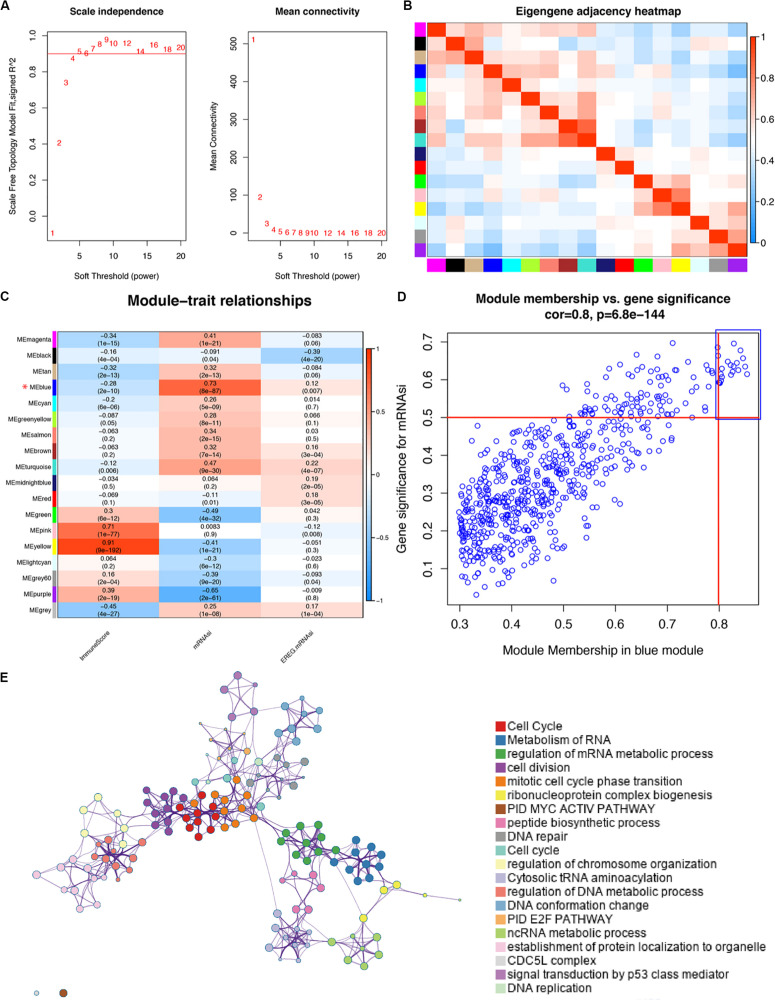
WGCNA of LUAD **(A)** Analysis of the scale-free fit index for various soft-thresholding powers (β) and the mean connectivity for various soft-thresholding powers. **(B)** Heatmap plot of the adjacencies in the eigengenes network. **(C)** Correlation between the module eigengenes and clinical traits of LUAD, including immune score, mRNAsi and EREG-mRNAsi. The correlation coefficient in each cell represents the correlation between the modules and traits, which increases in size from blue to red. The corresponding *p*-value is annotated. **(D)** Scatter plot of module eigengenes in blue module with cutoffs of cor.gene MM > 0.8 and cor.gene GS > 0.5 in order to select hub genes. **(E)** METASCAPE enrichment network visualization cluster in enrichment analysis of SRGs. Each circle node represents one term, and each color represents its cluster identity, showing the intra-cluster and inter-cluster similarities of enriched terms. Cluster annotations are shown in color code.

Based on the different gene characteristics of cancer tissues, we used WGCNA to construct a co-expression network to find modules and genes that were significantly related to mRNAsi and immune score. The power of β = 5 (scale-free *R*^2^ = 0.95) was chosen as the soft-thresholding parameter (Figure 2A) to ensure a scale-free network. Afterward, GS and MM were calculated for each gene, followed by the setting of the thresholds. The cut-off criteria of cor. GS > 0.5 and cor. MM > 0.8 were set to identify genes with relatively high correlation to the feature in the key module (Figure 2D). As shown in Figure 2C, the average link hierarchical clustering identified a total of 18 modules. The characteristics of immunity and stemness were represented by immune score and mRNAsi, respectively. The correlation between immune and stemness in each module were remarkably reverse. The heatmap plot of the adjacencies in the eigengenes network was shown in Figure 2B.

We selected the blue module as the key module, for it was most significantly related to stemness and immunity among the 18 modules. Because of our aim to explore the role of SRGs in treatment strategies and the potential application of SRGs as targets in drug design, we preferred to choose candidate genes that were highly up-regulated in the tumor tissue. The yellow module was excluded although it was also significantly related to both features. To assess the interaction of genes in the blue (key) module, we used the Metascape for enrichment analysis. Most of genes of the blue module have significant functional connections in cell proliferation and metabolism-related signaling pathways (Figure 2E).

Ultimately, 15 co-expressed genes were screened out, including CCNA2 (cyclin A2), CCNB1 (cyclin B1), CDC20 (cell division cycle 20), CDCA5 (cell division cycle associated 5), CDCA8 (cell division cycle associated 8), FEN1 (flap endonuclease 1), KIF2C (kinesin family member 2C), KPNA2 (karyopherin α2), MCM6 (minichromosome maintenance complex component 6), NUSAP1 (nucleolar and spindle-associated protein 1), RACGAP1 (Rac GTPase-activating protein 1), RRM2 (ribonucleotide reductase small subunit M2), SPAG5 (sperm-associated antigen 5), TOP2A (topoisomerases type IIα), and TPX2 (TPX2 microtubule nucleation factor).

### Validation of SRGs

**FIGURE 3:**
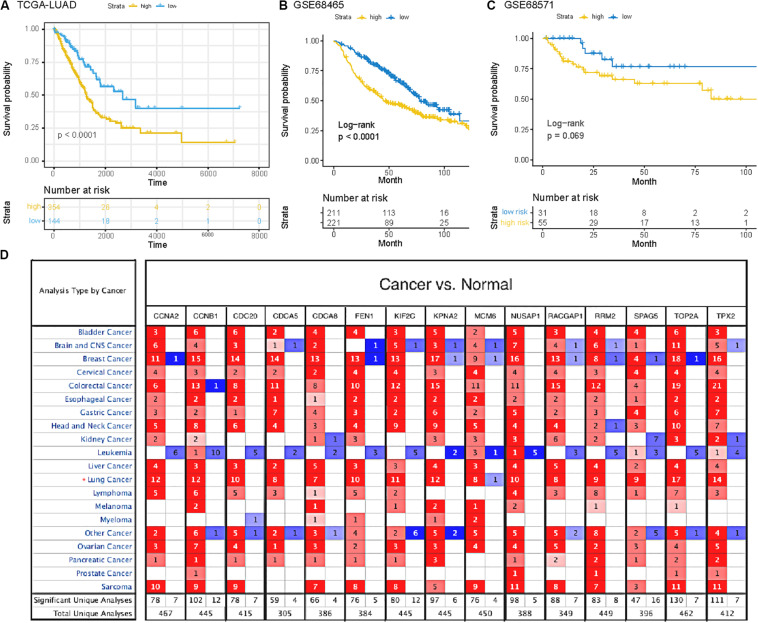
**(A)** Survival analysis of SRGs signature based on data from TCGA database. **(B,C)** Survival analysis of SRGs signature based on data from GEO database (GSE68465 and GSE68571). **(D)** The mRNA expression patterns of SRGs in several kinds of cancers. The differences of mRNA expression between tumors and normal tissues based on Oncomine database. The number in each colored cell represents the number of researches meeting these thresholds with color depth determined by the gene rank. The red cell suggests that the mRNA levels of target genes in tumor tissues are higher than that in normal tissues, while the cell in blue means the opposite.

To verify the potential value of the selected SRGs, we performed functional verification at both transcription and protein levels. Figure 3A demonstrated that the higher expression of 15 genes in LUAD cases was associated with decreased overall survival (*p* < 0.0001, HR = 1.90). Two independent cohorts (GSE68465 and GSE68571) obtained from the GEO database were used to verify the prognostic performance of the SRGs (Figures 3B,C). The results were consistent with the conclusion that SRGs were associated with poor prognosis. Compared with the mRNAsi (HR = 1.24) and immune score (HR = 0.70), the SRGs performed better in prognostic prediction, which were also more stable than stemness or immunity alone.

**FIGURE 4:**
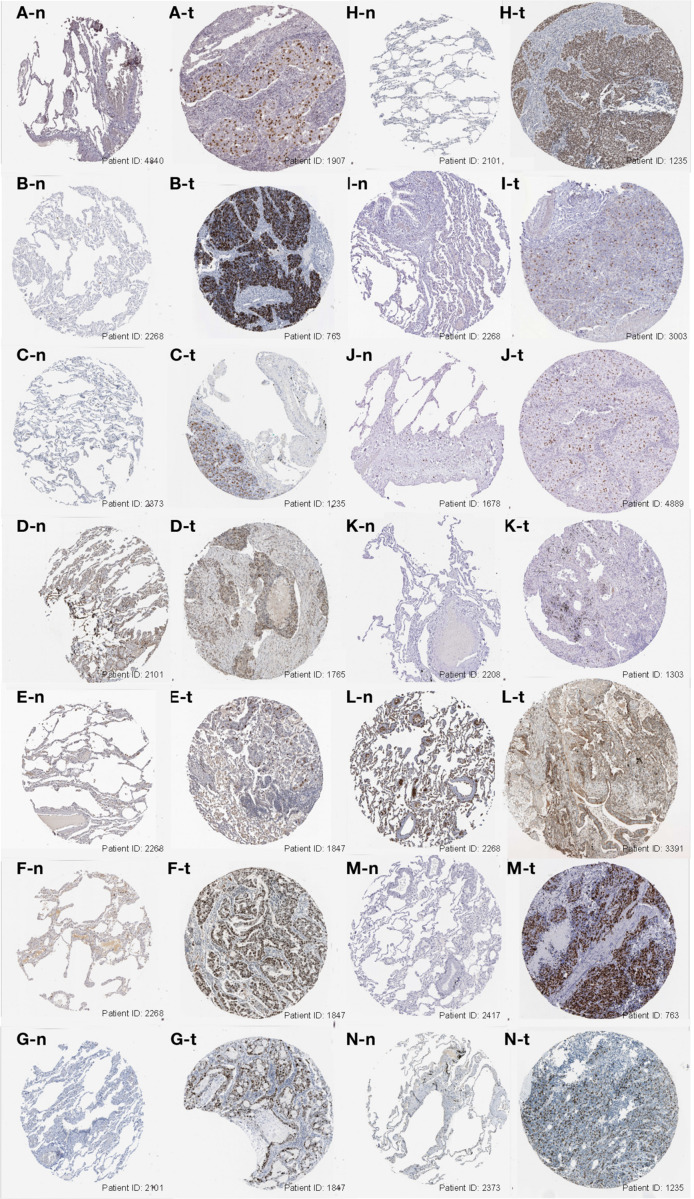
Immunohistochemistry images of the 15 SRGs (CCNA2, CCNB1, CDC20, CDCA5, CDCA8, FEN1, KIF2C, KPNA2, MCM6, NUSAP1, RACGAP1, RRM2, SPAG5, TOP2A, and TPX2) based on the Human Protein Atlas database were demonstrated in **(A–N)**. The level of protein expression was higher in tumor tissue (X-t) than that in normal tissue (X-n). *X* = A, B, and N (images of KIF2C were absent from the database).

Furthermore, we analyzed the genes expressed between tumor and normal tissues through the Oncomine database^3^ (Figure 3D). The threshold limits were set as follows: *p*-value, 1E-3; fold change, 1.5; gene level, 10%; data type, mRNA. As a result, the SRGs overexpressed not only in lung cancer, but also in many other types of cancers, especially breast cancer, colorectal cancer and sarcoma. In addition, the verification of protein level was analyzed using immunohistochemistry provided by the Human Protein Atlas (HPA) database. As shown in Figures 4A–N, compared with normal tissues, LUAD samples had significantly higher protein levels of SRGs.

**FIGURE 5:**
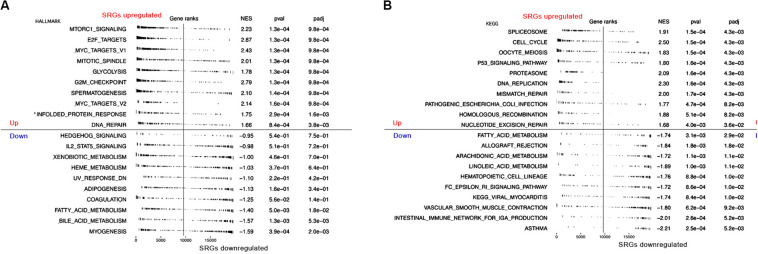
Gene Set Variation Analysis (GSVA) showing RNA sequencing (RNA-seq)-based immune and stemness signature evaluated in the context of SRGs sets representative for immunity, stemness and cancers. **(A)** GSVA analysis of HALLMARK database. **(B)** GSVA analysis of KEGG database.

**FIGURE 6:**
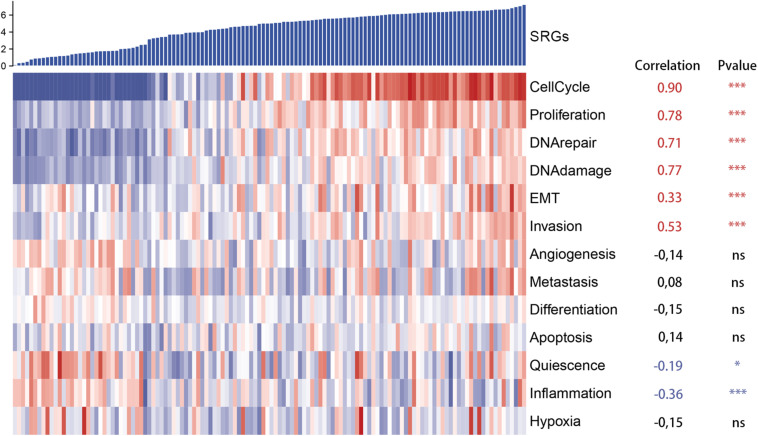
Heatmap of single cell RNA sequencing (scRNA-seq) of GSE69405 from the Gene Expression Omnibus (GEO). SRGs expression was positively correlated with several biological processions, including cell cycle, proliferation, DNA repair, DNA damage, epithelial-mesenchymal transition (EMT), which was demonstrated in red. In contrast, the inflammation and quiescence were in negative correlation with expression of SRGs, which was displayed in blue.

Moreover, to further assess SRGs related signaling pathways, we performed Gene Set Enrichment Analysis (GSEA) based on HALLMARK and KEGG database. As shown in Figures 5A,B, group with highly expressed SRGs was mainly enriched in several tumorigenesis and cell proliferation-related signaling pathways, such as MYC targets, cell cycle, P53 signaling pathways, whereas the group with low expressed SRGs was mainly enriched in immune regulatory pathways, including immune network for IgA production, IL2 STAT5 signaling, etc. In order to reduce the potential interference factors, we used pure cancer cells to explore the role of SRGs in tumor progression and the results demonstrated that SRGs expression was positively associated with several biological processions, including cell cycle, proliferation, DNA repair, DNA damage, and epithelial-mesenchymal transition (EMT). On the contrast, the inflammation and quiescence were in a negative association with SRGs expression (Figure 6).

### SRGs Expression With Immune Infiltration

**FIGURE 7:**
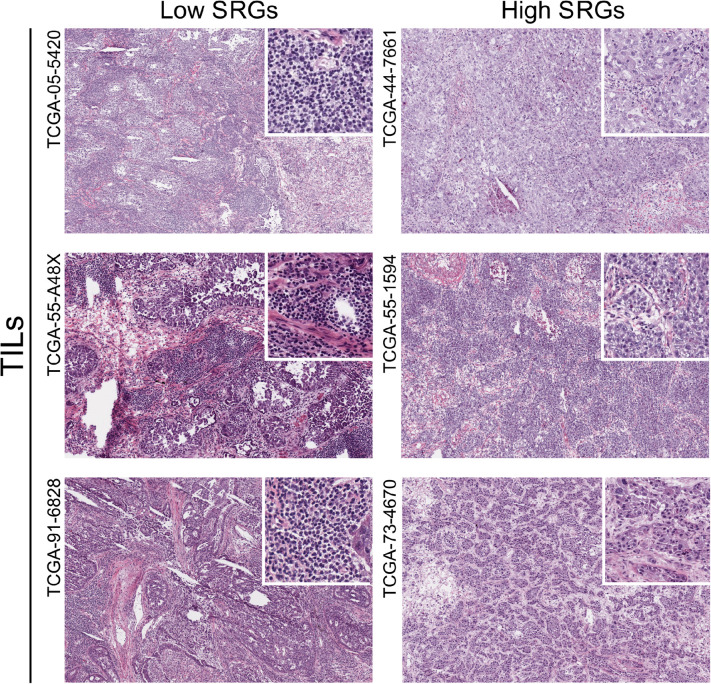
The hematoxylin and eosin (H&E) histopathological images of TILs in LUAD samples between high and low SRGs expression groups downloaded from the TCIA database.

To verify the correlation between stemness and immune infiltration, we used H&E histopathological images to compare the degree of immune infiltration with different expression levels of SRGs (high vs low). In Figure 7, the infiltration pattern of TILs in patients with high and low SRGs expression was obviously different. The level of TILs in high SRGs expression tissues could be lower than low SRGs group.

**FIGURE 8:**
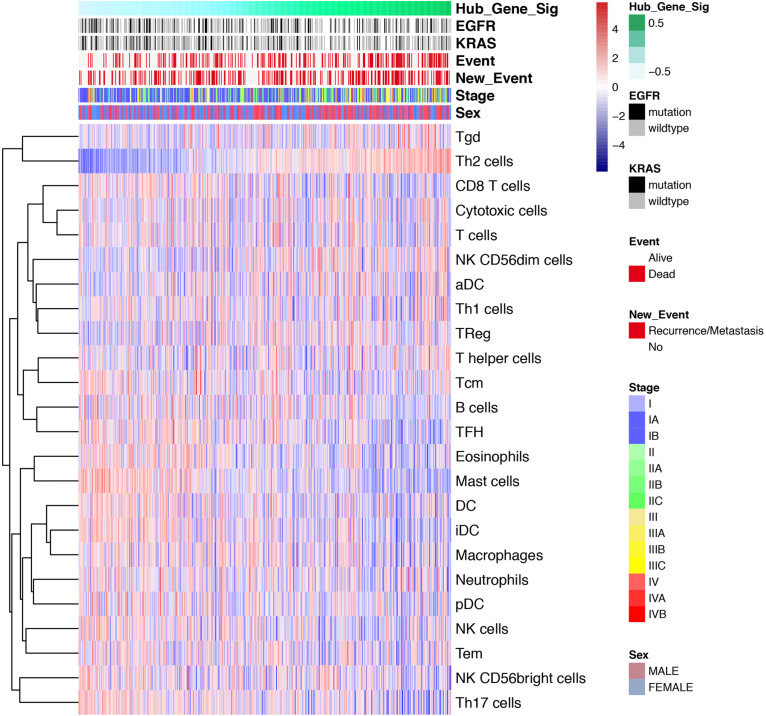
Single-sample Gene Set Enrichment Analysis (ssGSEA). Heatmap showing the correlation of transcriptome expression of SRGs with infiltration level of 24 immune cell types, mutation status of EGFR and KRAS, event, new event, stage and sex, as annotated in the right panel. The correlation coefficient represents the correlation between the event and the expression of SRGs. For example, the correlation coefficient of immunocyte infiltration and SRGs expression increases as the colors vary from blue to red in size.

Various immune cells in TME regulate the anti-tumor response through activation or suppression. Therefore, ssGSEA was performed to define the degree of up-down regulation of a particular component of TILs in a single sample. Our research involved 24 immunophenotypes, namely B cells, T cells, natural killer (NK) cells, dendritic cells (DCs), macrophages and other tumor-infiltrating lymphocytes. The results of the ssGSEA showed that the increase in infiltration status was negatively correlated with tumor progression (Figure 8), which was consistent with the above conclusion. Generally, most of the TILs including T cells, macrophages, DCs were negatively correlated with the expression levels of SRGs. However, the level of Th2 cell infiltration was positively correlated with SRGs expression.

### SRGs Expression With Clinical Therapeutic Strategies

**FIGURE 9:**
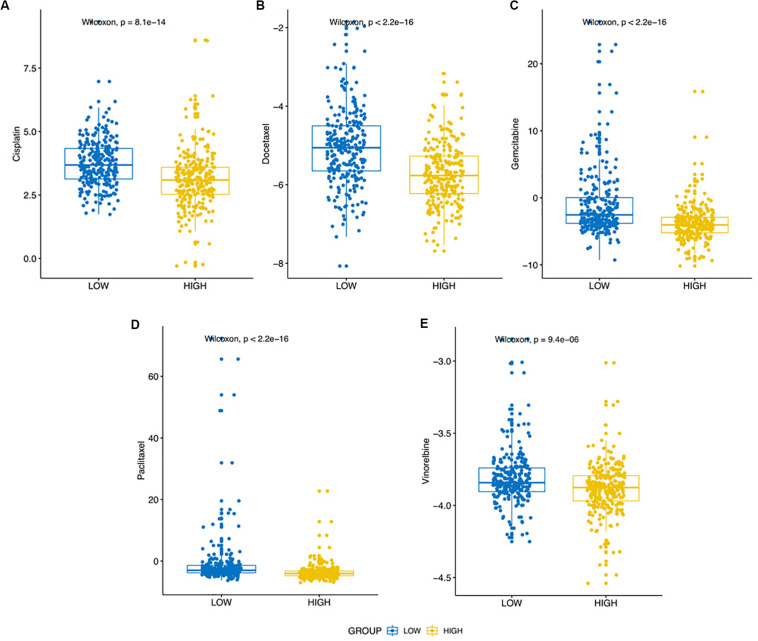
Differential putative chemotherapeutic response in high expression and low expression of SRGs groups. **(A–E)** The box plots of the estimated IC50 for five commonly applied clinical chemotherapeutic drugs including Cisplatin, Docetaxel, Gemcitabine, Paclitaxel, and Vinorelbine manifested the correlation between expression of SRGs and sensitivity to the chemotherapy.

Previous findings indicated that the expression of SRGs was significantly related to immune infiltration (negatively), stemness (positively) in LUAD samples. It indicated that the expression of SRGs played an important role in the tumor progression and immune infiltration, which could be a benefit to formulate clinical treatment strategies. To confirm this hypothesis, the “pRRophetic” R package was used to predict the therapeutic effects of five LUAD first-line chemotherapy drugs, including cisplatin, docetaxel, gemcitabine, paclitaxel, and vinorelbine. As shown in Figure 9, the half-maximal inhibitory concentration (IC50) of the higher expression SRGs group was significantly lower (Wilcoxon Rank Sum Test, *p* < 0.001).

**FIGURE 10:**
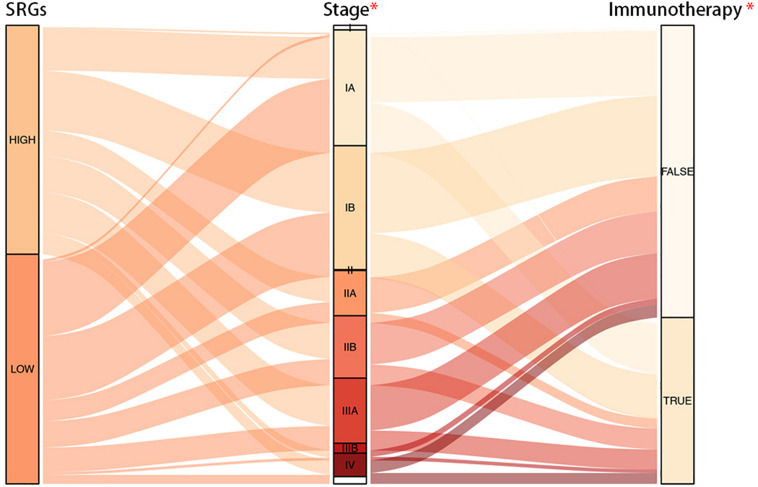
Alluvial diagram of SRGs expression with progression of LUAD and efficacy of immunotherapy. It was demonstrated that groups with low expression of SRGs were more sensitive to the immunotherapy.

The Connection Map (CMap) is a database related to small molecule drugs and mechanism of action (MoA), which was used to analyze the therapeutic value of small molecule inhibitors in LUAD. As a result, it was found that cytochrome P450 inhibitors, SIRT activators, phosphodiesterase inhibitors, and a total of eight inhibitors were potentially effective (Table 1). Through TIDE algorithm, we found that immunotherapy was predicted to relatively ineffective for patients with high SRGs expression (Figure 10).

**TABLE 1:** CMap and MoA analysis of samples with different SRGs expression.

**CMap name**	**Mean**	**Enrichment**	***p***	**MoA**
Resveratrol	−0.672	−0.78	0	Cytochrome P450 inhibitor, SIRT activator
Zardaverine	0.543	0.922	0.00004	Phosphodiesterase inhibitor
0173570–0000	−0.656	−0.799	0.00016	
5248896	−0.853	−0.988	0.00032	
Withaferin A	−0.681	−0.878	0.00052	
Pyrvinium	−0.665	−0.739	0.00066	
Trifluoperazine	−0.448	−0.479	0.0007	Dopamine receptor antagonist
Etoposide	−0.333	−0.842	0.00109	Topoisomerase inhibitor
Mevalolactone	0.462	0.917	0.00118	
Puromycin	−0.585	−0.839	0.00121	Protein synthesis inhibitor
Semustine	−0.307	−0.838	0.00121	
Parthenolide	−0.532	−0.835	0.00131	NFkB pathway inhibitor, Adiponectin receptor agonist
Lomustine	−0.393	−0.823	0.00187	
Loperamide	−0.404	−0.683	0.00262	Opioid receptor agonist
Irinotecan	−0.695	−0.885	0.00298	Topoisomerase inhibitor

*Table showing the enrichment score of each compound by Connectivity Map (CMap) database and mechanism of actions (MoA) for LUAD with p-value annotated.*

In summary, LUAD patients with high expression of SRGs tended to benefit less from immunotherapy, but in contrast, they could be more likely to receive positive results from chemotherapy.

## Discussion

The fact that chemotherapy and immunotherapy are not effective for some patients prompted us to search for the underlying molecular mechanism. In this study, after obtaining resources from the Internet and databases, we successfully identified the blue module and 15 genes which were significantly related to cancer stemness and immunity. After that, we further explored their relationship with immune infiltration patterns, functions in signaling pathways and clinical outcomes. Based on the findings, we suggested that the 15 SRGs could be used as biomarkers for treatment strategies design and prognosis prediction for LUAD patients.

Some previous studies have been published in this field, reporting genes related to stemness, which are associated with cancer recurrence, metastasis, resistance to treatment, and poor prognosis (Pan et al., 2019; Qin et al., 2020; Zhang et al., 2020; Zhao et al., 2020). For instance, a recent study identified 13 key genes enriched from KEGG pathway (Zhao et al., 2020). Four of which are the same as we found, namely, CCNA2, CCNB1, CDC20, and MCM6. The differences can be explained by gene co-expression (most of the genes identified in other studies were also in the key module of our study, but some of them have not been selected as the hub genes), samples diversity, random errors in the algorithm, or other unknown reasons. Compared with the previous studies, we adopted a similar process to screen candidate hub genes like them. However, we have certain advantages over these papers in further evaluation and exploration based on candidate genes. Our research not only explored the expression distribution, related pathways, and prognostic performance of these genes, but also explore the function of SRGs on TME and clinical therapies systemically.

A couple of SRGs reported by us were found to function in certain pathways and also other kinds of cancer according to previous studies. For example, FEN1 functions in multiple pathways of DNA metabolism and promotes tumor progression (Konsavage et al., 2012). KPNA2 is related to a kind of growth factor FGF1. It improves the functions of certain signaling pathways and ultimately increases the proliferation of cancer cells (Yamada et al., 2016). It has been reported that CCNB1 is a potential biomarker target in LUAD (Malta et al., 2018) and that CDC20 is highly expressed in LUAD tissues and may serve as a new type of prognostic biomarker (Liu et al., 2019). Apart from lung cancer, some SRGs are also related to other cancers. For instance, it was found that CCNB2 is overexpressed in a variety of tumors, including gastric cancer, bladder cancer, prostate cancer, uterine corpus endometrial carcinoma (Shi et al., 2016; Huang et al., 2017; Han et al., 2018; Shen et al., 2018).

In term of immunity, the overexpression of SRGs, which was positively related to cancer stemness, was associated with reduced immune infiltration. The findings were in accordance with previous studies (Limagne et al., 2016; Liu et al., 2018; Malta et al., 2018; Miranda et al., 2019; Liao et al., 2020). It has been reported that transcriptome-derived stromal and immune scores have implications for patient survival, metastasis and recurrence (Liu et al., 2018). A previous study also confirmed that the stemness index is related to immune microenvironment (Malta et al., 2018). Some SRGs, CCNA2, and CDC20, has demonstrated significant correlation with multipotent stromal cells, which are believed to have an impact on immune suppression (Bellayr et al., 2016). CDC20, CDCA8, and KIF2C were up-regulated in EBV-transformed lymphocytes and controlled several biological processes including immunity response (Dai et al., 2012). RACGAP1, TOP2A and some other genes co-expressed to regulate the immunity response in hepatocellular carcinoma (Drozdov et al., 2012). Since the immune infiltration of patients with high SRGs expression is reduced, it may explain the decline in the efficacy of immunotherapy.

Furthermore, the results of ssGSEA also showed that most TILs, such as T cells, Tregs, Th17, macrophages, and DCs, were negatively correlated with the expression levels of SRGs. Whereas, the infiltration level of Th2 cell was positively correlated with the SRGs. Th2 cell is one of the main T cell subtypes found in TME, which is known to help B cells and has the ability to produce anti-inflammatory cytokines (including IL-4, IL-5, and IL-13) (Mosmann and Coffman, 1989; Walker and McKenzie, 2018). Some studies reported that the cytokines secreted by Th2 are related to the suppression of the anti-tumor immune response (Kusuda et al., 2005; Ubukata et al., 2010), which is in accordance with our finding that the infiltration pattern Th2 cell was opposite to other TIL.

In addition, enrichment analysis showed that highly expressed SRGs were mainly involved in pathways related to mitosis and gene repair, including cell cycle, DNA redistribution, DNA repair, p53 signal transduction pathway, and MYC target. It indicates that those overexpressed genes are related to excessive cell proliferation is related to tumor progression. We found that the lower expression of SIRG was related to pathways related to immune response and metabolism, such as IgA production, IL2 state signaling, and immune network of hematopoietic cell lineage. The results indicate that the SRGs may play the role as a whole and are related to the progression of cancer and the efficacy of immunotherapy.

Compared with mRNAsi driven from the supervised machine learning, the SRGs performed better in prediction prognosis, and had greater interpretability and robustness, for there are plenty of algorithms that can be used to predict the therapies efficacy, such as TIDE used in our research. Based on the results, we provided an innovative treatment strategy which recommends chemotherapy or using certain small molecule inhibitors targeting SRGs in combination with immunotherapy for patients with high expression of SRGs to achieve better clinical outcomes.

In conclusion, 15 SRGs were identified as biomarkers of LUAD, which are not only suitable for prognosis, but also helpful to measure the treatment effect and tumor recurrence. The expression of SRGs is positively correlated with cancer stemness, but negatively related to anti-tumor immunity. However, there were some limitations in our study. Firstly, limited by the sample size and potential bias of the population included in our study, multicenter studies need to be supplemented. Besides, some of the findings can only result in information of correlation, not definite causation. Therefore, further molecular biology experiments and clinical verification are needed to confirm our discoveries.

## Data Availability Statement

Publicly available datasets were analyzed in this study. This data can be found here: TCGA database (https://portal.gdc.cancer.gov); GEO database (http://www.ncbi.nlm.nih.gov/geo/); Oncomine database (https://www.oncomine.org); TCIA database (http://www.cancerimagingarchive.net/).

## Author Contributions

HZ and JJ contributed to conception of the study and wrote the manuscript. XS performed the data processing. YH and HL edited the figures and table. JH revised the manuscript. XM gave final approval of manuscript. All authors agreed to be accountable for the content of the work.

## Conflict of Interest

The authors declare that the research was conducted in the absence of any commercial or financial relationships that could be construed as a potential conflict of interest.

## Footnotes

1 https://portal.gdc.cancer.gov

2 http://www.ncbi.nlm.nih.gov/geo/

3 https://www.oncomine.org

4 http://www.cancerimagingarchive.net/

5 http://metascape.org

6 https://www.cancerrxgene.org/

7 https://clue.io/
